# Correction: Community participation and technological innovation: Baseline qualitative insights to inform a five-year cohort on drone-based dengue surveillance in Malaysia

**DOI:** 10.1371/journal.pntd.0014316

**Published:** 2026-05-13

**Authors:** Rahmat Dapari, Safiyeh Tayebi, Ana Lorena Ruano, Timothy C. Guetterman, Seok Mui Wang, Siti Hafizah AB Hamid, Sohel Rahman, Jürgen Pilz, Nazri Che Dom, Ubydul Haque

The tenth author, Ubydul Haque, is incorrectly noted as the corresponding author. The correct corresponding author is Ana Lorena Ruano. Ana Lorena Ruano’s email address is: Ana.Lorena.Ruano@uib.no.
