# Supplementary material for: Community participation and technological innovation: Baseline qualitative insights to inform a five-year cohort on drone-based dengue surveillance in Malaysia
Source: PLoS Negl Trop Dis. 2026 Mar 12;20(3):e0013979. doi: 10.1371/journal.pntd.0013979 (PMC12998943; doi:10.1371/journal.pntd.0013979)
Supplement: Attachment — Submitted filename: Rebutal letter with response to editor 12 Nov.pdf [file pntd.0013979.s003.pdf]

1) Please ensure that the CRediT author contributions listed for every co-author are completed accurately and in full.
At this stage, the following Authors/Authors require contributions: Rahmat Dapari, Safiyeh Tayebi, Ana Lorena Ruano, Timothy C. Guetterman, Seok Mui Wang, Siti Hafizah, Sohel Rahman, Jürgen Pilz, Nazri Che Dom, and UBYDUL HAQUE. Please ensure that the full contributions of each author are acknowledged in the "Add/Edit/Remove Authors" section of our submission form.
The list of CRediT author contributions may be found here: https://journals.plos.org/plosntds/s/authorship#loc-author-contributions

Our response

All are correct

2) Please provide an Author Summary. This should appear in your manuscript between the Abstract (if applicable) and the Introduction, and should be 150-200 words long. The aim should be to make your findings accessible to a wide audience that includes both scientists and non-scientists. Sample summaries can be found on our website under Submission Guidelines:
https://journals.plos.org/plosntds/s/submission-guidelines#loc-parts-of-a-submission

Our response

Author summay has been provided (pink highlight)

3) Please upload all main figures as separate Figure files in .tif or .eps format. For more information about how to convert and format your figure files please see our guidelines:
https://journals.plos.org/plosntds/s/figures

Our response

All Figure has been upload separetly

4) Please upload a copy of Figures "1, and 2" which you refer to in your text on page in your Manuscript. Or, if the figure is no longer to be included as part of the submission please remove all reference to it within the text.

Our response

All Figure has been upload separetly

5) We have amended your Competing Interest statement to comply with journal style. We kindly ask that you double check the statement and let us know if anything is incorrect.

Our response

Correct

6) In the online submission form you indicate that your data is available from a co-author on this manuscript.
According to our Data Policy, data must either be included with the submission, be placed in a public repository,
or available from a third party contact point who must not be an author on the manuscript. Please revise your data statement to a non-author institutional point of contact, such as a data access or ethics committee, and send this to us via return email. Please also include contact information for the third party organization (this contact should be a central address and not a named person), and please include the full citation of where the data can be found.

Our response

Data availability has been revised

All data supporting the findings of this study are fully available within this manuscript.

Author summary: findings are important for improving the future efficiency of container surveillance in areas where dengue is endemic. The authors emphasise that drone-based surveillance is defined by local social and cultural context, which is an important finding for these types of potentially invasive technologies.

PNTD-D-25-00650
Community Participation and Technological Innovation: A Prospective Cohort Study on Drone-Based Dengue Surveillance in Malaysia

PLOS Neglected Tropical Diseases

Editor's comments
1. Comments: Throughout the manuscript, the authors continually refer to the use of drones for dengue control or dengue surveillance. It is even in the title of the manuscript. The drones being described in this manuscript and references provided are not being applied in dengue control; *they are being employed in container surveillance for mosquito vectors of dengue (sometimes referred to as mosquito breeding site monitoring in the manuscript)*. It is important that this difference is made clear throughout the majority of the manuscript and removed from the title. The only way drones can be talked about in the context of dengue is to improve outcomes of dengue management or dengue control (i.e., improved efficiency of surveillance improves management outcomes).

Response: We agree that the distinction between vector surveillance and dengue surveillance is vital for the manuscript's precision. Accordingly, we have revised the terminology throughout the manuscript, including the title, abstract, and keywords, to specify that the drones are used for 'surveillance of mosquito breeding sites' as a component of a broader dengue control strategy.
The revised title: **Community Participation and Technological Innovation: Baseline Qualitative Insights to Inform a Five-Year Cohort on Drone-Based Dengue Surveillance in Malaysia**

2. Comments: Use of the term drone interventions and drones as mosquito control tools implies that the drones are acting to manage dengue, when, in the case described in the manuscript or in the references, they are being used for passive monitoring. Again, please consider the use of a passive term such as surveillance.

Response: Specifically, the terminology of 'surveillance', 'strategies', and 'surveillance technologies' has been adopted, clarified as 'vector surveillance' or 'surveillance of mosquito breeding sites,' to accurately describe this passive activity.

Introduction
3. Comments: It would benefit the manuscript for readers to know more about the program under which the drones were being used for surveillance in Selangor. What are the objectives of the larger study? To improve the efficiency of mosquito control? Is container surveillance being validated as was done in the studies referenced?

Response: We have added the following text: "Currently, drone-based surveillance is not part of Selangor's official dengue control programmes. This prospective study was therefore designed to assess community and stakeholder perceptions, acceptance, and social feasibility before launching a larger cohort study. The findings will inform methodological development and support potential integration of drone technology into future pilot vector control programmes, particularly to enhance container surveillance efficiency," in the introduction section. ***Please see page 5, lines 131-135.***

Findings
4. Comments: Please provide more context for the readers on what communities you are referring to. Malaysian communities? Findings will be relevant to the culture in which they are being studied, so this needs to be emphasised throughout, especially in the discussion. It is also a limitation of the findings that needs to be discussed in the limitations section.

Response: We have integrated this context more thoroughly into the Discussion. "While this study found high community acceptance in Malaysia, we recognize that a successful, long-term implementation would require a comprehensive operational framework. Therefore, for a future study or

pilot program to proceed, it will be critical to develop and transparently communicate a Standard Operating Procedure (SOP). This SOP must address key governance and privacy issues, including securing necessary approvals from aviation authorities, establishing clear data governance protocols for image storage and access, and operationalizing a consistent community notification system. Addressing these practical concerns will be essential for translating the high acceptance found in this study into sustained public trust and participation." **Please see page 16, lines 436 – 443**.

Furthermore, we have now included a paragraph in the Limitations section: "This study was conducted within the socio-cultural context of multi-ethnic, urban communities in Selangor, Malaysia, where perceptions and high acceptance were shaped by local norms and strong trust in public health authorities. While these dynamics are context-specific, the findings provide valuable insights that can guide adaptation of drone-based surveillance approaches in diverse settings with different community–government relationships and views on technology". ***Please see Page 17, lines 451 – 455.***

5. Comments: Page 10: Please explain in further detail how drones can be deployed with the specific objective of "identifying new clusters based on reported dengue cases" or otherwise removed.

Response: The sentence has been removed.

6. Comments: Page 10: You mention drone use "framed around rapid detection and targeted response". The majority of what has been discussed to this point has been around surveillance. In this context, what do you mean by rapid detection and response?

Response: We have revised it to better align with the manuscript's focus on surveillance. The sentence now clarifies that 'rapid detection' refers to the timely identification of potential mosquito breeding sites by the drone, and 'targeted response' refers to the subsequent, more efficient deployment of public health vector control teams to those specific locations. ***Please see page 10, lines 260 – 262.***

Discussion

7. Comments: Second paragraph needs the font corrected and integrated into one of the other paragraphs before or after this one sentence.

Response: Correction done.

Limitations

8. Comments: The use of the wording "potential breeding sites" is critical in this manuscript, as drones are not as useful as active monitoring when samples are taken from containers for identification. Potential mosquito breeding sites still need to be validated by field staff on the ground. Detection of potential breeding sites will be more efficient in dengue endemic areas to direct mosquito control, but not for those outside endemic regions that do not utilise regular insecticide spraying programs.
Please include the limitation that findings may only be applicable to the cultural setting in which they were observed.

Response: We have added limitations. ***Please see Page 16, 17, lines 445 – 460.***

Conclusion

9. Comments: Please include Malaysia as the country in which the study was undertaken, after the use of Selangor.

Response: We have included. Thank you.

Specific Issues

10. Comments: Figure 2: fonts are too small to read.
Response: figure has been revised

11. Comments: First use of *Aedes aegypti* in full, then use *Ae. aegypti* both with italics.

Response: We have revised the entire manuscript.

Reviewer #1
12. Comments: Please provide more context for the readers on what communities you are referring to. Malaysian communities? Findings will be relevant to the culture in which they are being studied, so this needs to be emphasized throughout, especially in the discussion. It is also a limitation of the findings that needs to be discussed in the limitations section.

Response: We are referring to Selangor communities. Further explanation has been added in the limitation section. ***Please see Page 17, line 452.***

13. Comments: Page 10: Please explain in further detail how drones can be deployed with the specific objective of "identifying new clusters based on reported dengue cases" or otherwise removed.

Response: The sentence has been removed.

Discussion
14. Comments: Second paragraph needs the font corrected and integrated into one of the other paragraphs before or after this one sentence.
Response: Revised. Thank you.

15. Comments: It would benefit the manuscript for readers to know more about the program under which the drones were being used for surveillance in Selangor. What are the objectives of the larger study? To improve the efficiency of mosquito control? Is container surveillance being validated as was done in the studies referenced?

Response: We have revised the Introduction to provide the necessary context for the reader. The manuscript now includes a statement clarifying that the drone surveillance is not part of a current official program in Selangor. We explain that this study was specifically designed as a prospective assessment to gauge community feasibility and inform the objectives, such as improving the efficiency of mosquito control for a potential future pilot program.

16. Comments: Throughout the manuscript, the authors continually refer to the use of drones for dengue control or dengue surveillance. It is even in the title of the manuscript. The drones being described in this manuscript and references provided are not being applied in dengue control; they are being employed in container surveillance for mosquito vectors of dengue (sometimes referred to as mosquito breeding site monitoring in the manuscript). It is important that this difference is made clear throughout the majority of the manuscript and removed from the title. The only way drones can be talked about in the context of dengue is to improve outcomes of dengue management or dengue control (i.e., improved efficiency of surveillance improves management outcomes).

Response: Please see the response to the editor's comments number 1.

17. Comments: Use of the term drone interventions and drones as mosquito control tools implies that the drones are acting to manage dengue, when, in the case described in the manuscript or in the references, they are being used for passive monitoring. Again, please consider the use of a passive term such as surveillance.

Response: Please see the response to the editor's comments number 2.

Reviewer #2:
18. Comments: Formatting: Remove the unexpected page break after the Introduction; ensure consistent section headers. Additionally, there are no line numbers in the pages, so I'm not providing specific line changes, since that would have taken too much time to reference (page, paragraph, and line within paragraphs).

Response: We have added page and line numbers. Thank you.

19. Comments: Demographics table: The current Table 1 appears garbled; please present as a clean table with clear headers and totals (n and %).

Response: We have added n and % and added a new title “Distribution of respondents by gender, age group, occupation, and education.”

20. Comments: References: Replace Wikipedia citations (e.g., for Selangor) with official government or statistical sources. Check that all claims in the framework and discussion cite primary literature (several sentences mention studies but list only a single reference). There are only 26 references, almost as few as a short report (20 max).

Response: We have replaced the reference with the Department of Statistics Malaysia.
We appreciate the reviewer’s suggestion to check all the references in the discussion section. However, due to the unique nature of community engagement in a prospective cohort study, there are not many relevant published studies that we can cite. Thank you.

21. Comments: Figures: Ensure vector exports for diagrams and color-blind-safe palettes (e.g., viridis-family) where possible.
Response: revision done

Reviewer #2

Major comments
22. Comments: Title/design mismatch (prospective cohort vs qualitative cross-sectional)

Issue: The title and some sections describe a “prospective cohort study,” yet the methods and results report semi-structured interviews (IDIs/KIIs) at a single time point with thematic analysis. Re-title and reframe as a qualitative formative study nested within or to inform a planned five-year cohort/surveillance program. You need to clearly state that these findings are qualitative baseline perceptions rather than cohort outcomes.

Response: We thank the reviewer for the suggestion. The new title is “*Community Participation and Technological Innovation: Baseline Qualitative Insights to Inform a Five-Year Cohort on Drone-Based Dengue Surveillance in Malaysia*”.

23. Comments: Sampling and recruitment require much greater clarity
Issue: You state that 506 households were approached and 480 IDIs completed (60 per locality × 8). You also mention “typical case sampling” and that adjacent households were contacted if a head was unavailable. It is unclear whether participation was random, purposive, convenience, or a hybrid; who exactly was eligible/responded (e.g., “household head” vs “any adult”); and what inclusion/exclusion criteria were used.
I would recommend a more transparent sampling and recruitment subsection: define sampling strategy (e.g., typical case purposive sampling), sampling frame, approach procedures, respondent definition, and replacement rules. State who was interviewed in households (household head vs any adult), the number approached, refusals, and the acceptance rate per site.

Response: We have rewritten this section as “A total of 506 households were initially approached across the eight study localities (targeting approximately 60 per site). Households were identified using a typical case purposive sampling approach to capture diversity in demographic and socioeconomic characteristics within each locality [15]. The intended respondent was the household head; if the household head was unavailable or declined, an adjacent household was approached as a replacement. In cases where the household head could not participate, another adult member was interviewed. Of the 506 households approached, 480 consented and completed in-depth interviews, corresponding to an overall acceptance rate of 95%. ***Please see Page 7, lines 177 - 183.***

24. Comments: More importantly, there is no explanation for why 480 IDIs and 6 KIIs were appropriate. For qualitative work, sample size is typically justified by saturation rather than power. Please provide a clear definition of why these sample sizes were enough. If pragmatic factors dictated the number (e.g., one per household in a baseline cohort), say so, and discuss implications. For KIIs, justify why n=6 was sufficient across stakeholder domains.

Response: We thank the reviewer for this important observation regarding sample size justification. In this study, the number of in-depth interviews (IDIs) was pragmatically determined by the baseline design of the larger five-year cohort program. Specifically, one respondent per household was interviewed during enrollment, resulting in 480 IDIs across the eight localities (approximately 60 households per site). Thus, the IDI sample size reflects the cohort's baseline recruitment strategy rather than being determined by a priori notions of saturation. We recognize that this differs from purely inductive qualitative research, where sample size is typically guided by thematic saturation. In our case, saturation was nevertheless reached, as we generated consistent themes across sites, and no substantively new issues were identified in later interviews. We have revised the text to clarify this rationale and its implications for interpretation.

For the key informant interviews (KIIs), the sample (n=6) was selected to represent the major stakeholder groups directly relevant to dengue control and the planned surveillance program (local health authorities, vector control staff, community leaders, and municipal representatives). While limited in number, these KIIs were designed to provide complementary contextual insights rather than serve as the sole basis for thematic analysis. The small number was also shaped by pragmatic considerations, including the availability of relevant stakeholders. We have clarified this justification in the revised manuscript and explicitly noted the implications for the transferability of findings.

Please see page 7, lines 184 – 188, page 8, lines 197 – 199.

25. Comments: Timelines and interview duration are inconsistent

Issue: KIIs are reported as 45–60 minutes and then 25–40 minutes in the next sentence; household IDIs' timelines are not clearly specified (May–July 2024 is later provided). Harmonize and report exact fieldwork windows for IDIs and KIIs, and consistent duration ranges for each. Provide the date range for household interviews, not only KIIs.

Response: We have clarified that 'Household IDIs lasted 25–40 minutes', and KIIs ranged from 45 to 60 minutes in length. ***Please see page 7, line 188, and page 8, line 200.***

26. Comments: Conceptual framework needs citations and positioning
Issue: The second line of the Conceptual Framework section refers to a logic model without citing foundational frameworks; the five-stage "inputs→impacts" model is common but currently under-referenced. Add appropriate citations to established implementation/logic-model frameworks (e.g., logic models used in public health interventions, implementation frameworks). Also, clarify how this framework guided instrument design, analysis, and interpretation (not just presented as a figure). The introduction feels as if more references should be added, since the author mentions on several occasions "studies," but sometimes does not provide a citation or just cites 1 study.

Response: We thank the reviewer for this constructive comment. We agree that the logic model and conceptual framework require clearer grounding in established implementation and public health frameworks. In the revised manuscript, we now cite foundational sources on logic models and implementation science (e.g., W.K. Kellogg Foundation Logic Model Development Guide; CDC Framework for Program Evaluation in Public Health; RE-AIM and CFIR frameworks). We have also clarified how the five-stage "inputs→impacts" model was adapted and operationalized in this study. Specifically, the framework guided the design of our data collection instruments (by structuring questions around resources, processes, and anticipated outcomes), our coding and thematic analysis (by organizing themes along the model's stages), and our interpretation of findings (by mapping perceptions and challenges to different stages of the pathway). Additionally, we have expanded citations in the introduction where studies are referenced without adequate support, ensuring a stronger connection to existing literature.

The following revised conceptual framework has been added
"The framework (Figure 1) integrates insights from community-based vector control, technological adoption in public health, and drone-assisted surveillance. To structure this, we applied a five-stage logic model: *Inputs, Processes, Outputs, Outcomes, and Impacts*, commonly used in public health intervention planning and evaluation [6, 14, 15]. This approach is consistent with established implementation frameworks such as RE-AIM and the Consolidated Framework for Implementation Research (CFIR), which emphasize linking resources and activities to measurable outcomes and long-term impacts [13, 16, 17].

The framework captures the technical, organizational, and social dimensions essential for sustainability. It integrates technological elements (drone mapping, AI analytics), community engagement strategies, and institutional collaboration mechanisms, as highlighted in previous studies on trust, compliance, and adoption in public health interventions [18]. Beyond providing a conceptual figure, this framework directly informed the study design: instruments were developed to probe inputs (resources, infrastructure, community readiness), processes (household participation, institutional roles), and anticipated outputs/outcomes (acceptability, feasibility, and perceived benefits). During analysis, themes were coded and organized along the five stages of the model, which also structured the interpretation of baseline perceptions. Thus, the framework not only presents a visual model but also serves as a guide for instrument development, analytic strategy, and interpretation of findings.
Please see page 6, line ……

27. Comments: Qualitative methods' rigor (trustworthiness) needs strengthening

Issue: Thematic analysis is described, but reporting is thin on reflexivity, coder training, intercoder agreement/consensus procedures, codebook development, data management, and steps taken to enhance credibility (member checking, triangulation), dependability (audit trail), and confirmability. Report according to COREQ or SRQR (PLOS encourages established qualitative reporting standards). Add: interviewer backgrounds/training, language/translation and back-translation procedures, how the codebook was developed/refined, how discrepancies were resolved, whether saturation was assessed (and how) for the very large number of IDIs (n=480), and any triangulation or member-checking conducted.

Response: qualitative methis on the interviewer, data collection and data management has been added

28. Comments: Analytic transparency and use of descriptive data

Issue: Findings are presented narratively with quotes, but readers of PLOS NTD will expect at minimum participant characteristics (counts/percentages) and, where appropriate, careful indication of how prevalent key themes were (without over-quantifying). Provide a clean Table 1 with respondent demographics by site (n, %; e.g., gender, age bands, occupation, education) and KII roles. In the Results, replace vague frequency terms (e.g., “frequently”, “many”) with n/N (%) where defensible (e.g., “privacy concerns were raised by 42/480 [8.8%] respondents”), while remaining faithful to qualitative norms.
Response:
We appreciate the reviewer’s constructive feedback. In response, we have added a new Table 1 summarizing participant characteristics with counts and percentages. This table presents the demographic diversity across the 480 household respondents and 6 key informants, clarifying sample composition by group.
We have also revised the Results section to improve clarity and precision by replacing vague frequency terms with quantitative expressions where defensible.

29. Comments: Data availability statement (PLOS policy)

Issue: “All data can be obtained from the corresponding author” does not comply with PLOS’s data policy for published articles. Deposit a minimal de-identified dataset suitable for qualitative research (e.g., codebook, theme matrix, anonymized quote compendium, and de-identified participant characteristics) in a stable repository (e.g., Dryad, Zenodo, figshare) and update the Data Availability Statement accordingly.

Response: This study is based on qualitative data collected from households and stakeholders in Malaysia. Due to ethical and legal restrictions under HIPAA and the conditions of Institutional Review Board (IRB) approval, the data cannot be shared publicly. The narratives and contextual details contain potentially identifiable information that cannot be fully anonymized without compromising participant confidentiality. At the time of consent, participants were informed that their data would be used solely by the study team, and external sharing was not permitted. Therefore, in order to uphold our ethical obligations and participant trust, no portion of the dataset (including transcripts, codebooks, or de-identified excerpts) can be made publicly available. All relevant methodological details, including

sampling, recruitment, coding framework, and analytic procedures, are provided within the manuscript to ensure transparency. We apologized to the reviewer.

30. Comments: Governance and regulatory context
Issue: You discuss trust and SOPs, but provide little about local regulatory approvals for drone flights (aviation/privacy) and data governance (who stores, who accesses, retention, anonymization). Add a short paragraph on operational approvals, data security, image handling (faces/license plates blurred or not recorded), altitude limits near residences, and how community notification is operationalized (e.g., posted schedules).

Response: We agree that a detailed operational plan is beyond the scope of this prospective study. We have revised the Discussion section to frame these important considerations as a recommendation, stating that any future study will need to establish a clear protocol covering regulatory approvals, data governance, and community notification to maintain public trust. ***Please see page 17, lines 464 – 471.***

31. Comments: Limitations: Add potential social desirability bias, translation/interpretation limitations, and context-specific generalizability.

Response: The limitation has been rewritten. Please see lines 473 – 488.

32. Comments: Please review that all acronyms are being spelled out prior to use, specifically for KII.

Response: Please see page 8, line 210.

33. Comments: Figure quality, accessibility, and map/satellite citation: Figures are pixelated; Figure 2's inset map is too small; color-blind accessibility is not addressed; and any satellite/aerial basemaps must be properly cited (Google/Bing/OSM have explicit requirements).

Response:
We thank the reviewer for noting this issue. All figures have been replaced with high-resolution versions.

34. Comments: Household 'survey' vs interview terminology: The manuscript sometimes calls the household component a "survey," but methods describe semi-structured IDIs. Use consistent terminology (e.g., "household in-depth interviews"). If a structured questionnaire was also administered, describe its content, type (closed/open items), and how those data were analyzed (basic descriptives), or remove "survey" language.

Response: We apologize to the reviewer. Here, we did not find 'survey' in the manuscript. All questions were open-ended.

Reviewer #3
This article offers interesting evidence from a qualitative sense on the households and stakeholder perceptions of drone-based dengue control in Selangor. Whilst the findings from the study indicated and discussed substantial issues and concerns, there are some aspects of this article in need of further clarification to ensure its logical flow and consistency. Here are some suggestions for the authors' kind consideration:

- Abstract

35. Comments: There is a need to include the keyword 'prospective cohort study' in the Background statement of: "To inform a five-year automated…' as mentioned in the article for consistency.

Response: We have incorporated. Thank you. ***Please see page 2, lines 32 – 34.***

36. Comments: Also, in the conclusions section, there is a thematic like analysis done that highlighted community motivation, collective responsibility, …trust, transparency, and consistent communication…and the success of the project. Please ensure consistency of what is written in the abstract to be similar to what is described in the article sections.

Response: conclusion has been revised

- Introduction

37. Comments: There are many statements made in the article that are not supported with the relevant references, for instance, on page. 4-5:

'….technological promise of drones is widely acknowledged, their successful integration into health systems is far from straightforward….."

'…often implemented without the full integration of local knowledge, institutional support, or community input.'
'Selangor, Malaysia, has consistently battled recurring dengue outbreaks'
Statements that are based on factual instances need to have statistical evidence, together with the relevant references.

Response: We have added references. We have also rewritten the statement with data "Selangor, Malaysia, has consistently battled recurring dengue outbreaks, accounting for 53.2% of national cases between 2015 and 2021, and enduring the highest incidence rates among all states, with up to 1,115 dengue cases per 100,000 people in 2019 [14, 15].
Please see page 4, lines 101 – 102; 117 – 119; 120 – 122.

38. Comments: In the conceptual framework, it was stated that the framework integrates '…insights from the literature on community-based vector control, technological adoption in public health, and drone-assisted surveillance. Where is the summary of the literature review conducted for this article?

Response: the statement has been revised

39. Comments: As for Figure 1, the research conceptual framework, was it modified or adapted from other sources? Please indicate if so.
Similarly, for the study setting, statements that are factually driven, such as '…rapid urbanization, high population density and high-rise buildings that create ideal breeding grounds for Aedes mosquitoes.' should be supported with references.

Response: We have added a citation. ***Please see lines 187 – 188.***

40. Comments: In Figure 2, the study setting map given is difficult to read and see the exact location. Consider revising this map.

Response:
We thank the reviewer for noting this issue. All figures have been replaced with high-resolution versions

41. Comments: There was no justification as to why the eight localities were chosen to represent Selangor. And there is usage of acronyms that are not explained before being used, such as IDI, KII, ST, UH, and ALR. Consider providing the explanation.
Response: We appreciate the reviewer's observation. The eight localities were purposively selected to represent Selangor's geographical and demographic diversity, including both urban and peri-urban settings. This approach allowed us to capture a broad range of environmental, socio-economic, and educational conditions that characterize the state. Given the study's total sample size of 480, selecting multiple sites maximized the geographic spread and enabled meaningful comparison across different community contexts, thereby reflecting Selangor's urban heterogeneity and variation in socio-economic backgrounds.
We have already elaborated IDI, KII, ST, UH, and ALR in the manuscript. ***Please see page 9, lines 230 and 231.***

42. Comments: In terms of the layout, the writing and empty spaces in between each section, as observed from Data Analysis on pg. 8 and 9, Findings. Please rectify accordingly.
Response: revision done

43. Comments: As for this results section, the qualitative style is not fully explored; often, the authors seem to use expressions such as ‘many in the community appear to view….’. There is a need to be objective and specific, even though you are describing the findings in a qualitative manner. Since the authors collected household data of 506 respondents, how were the demographic results, as stated in Table 1, assisted in the presentation of this Findings section? In fact, the authors seem to give a blanket statement of findings for the eight localities. Even though the author did mention it on page. 7 ‘ensuring diversity in urban heterogeneity and socioeconomic and educational backgrounds.’ However, there was no clear attempt made by the authors to provide this diversity in terms of the households' and stakeholders' perceptions and acceptance towards informing them on the implementation of this five-year automated drone surveillance program.

Response:

We thank the reviewer for this important observation. We have revised the Findings section to better integrate demographic diversity into the narrative. Specifically, we clarified how variations in gender, occupation, and education levels informed differences in perception and acceptance across the eight localities.

44. Comments: Whilst it is interesting that the authors used quotes from the respondents and key informants to support each main sub-section of the findings. It is still important to address the importance of eight localities in highlighting what the findings were in terms of their similarities and differences. The authors should also incorporate 1-2 paragraphs before presenting each of the sub-sections, as stated, there are four sub-sections. What are they? Themes? Elements? Dimensions? Unclear.

Response: we did not collect the localities characteristic for comparison. We have put this in our limitation for future study

45. Comments: Also, there was no specific mention of the program of this prospective cohort study in detail, particularly to describe the five-year automated plan. What is and who is involved? These pertinent points were not mentioned. How was the prospective cohort study conveyed to the households? Missing points to provide a logical understanding as to how the findings were collected from the households and stakeholders.

Response: We appreciate the reviewer’s valuable feedback. The present manuscript is focused on reporting the findings, while the detailed description of the prospective cohort program, including the five-year automated plan, study procedures, stakeholders involved, and methods of engagement with households, will be published separately in a dedicated study protocol paper. As such, the full methodological and programmatic details are beyond the scope of this manuscript, but will be made publicly available through the forthcoming protocol publication.

46. Comments: Having a clear explanation of what the sub-section described in the findings would help to reiterate in the discussion, although they were mentioned as ‘key dimensions. Please clarify this. Also, there are vague statements made on the page. 14 where authors mentioned ‘As previous research on vector control has shown’…which one?

Response: Here, the comment refers to previous research, which references 17, 18, and 19.

- Conclusion

47. Comments: In this section, the authors summarized what has been said in the findings and discussion sections. Perhaps the authors could consider synthesizing the main points and how to bring these points forward from the study.

Response: We thank the reviewer for the suggestion and rewrote the conclusion.

“This study highlights how communities in Selangor perceive dengue as an enduring health challenge that disrupts daily life and livelihoods, reinforcing the need for continuous and proactive interventions. Residents demonstrated heightened awareness of the disease and recognized the importance of sustained surveillance, which created openness toward innovative approaches such as drone-based monitoring. Importantly, participants emphasized that drones should be deployed strategically and efficiently, targeting high-risk areas rather than conducting unfocused flights, and that clear communication of their purpose and limitations is essential to maintain trust. While most viewed drone surveillance as non-invasive when focused on environmental factors, some concerns about privacy and

discomfort near residential spaces indicate the importance of transparent guidelines, operator training, and community engagement. Support for participation was largely motivated by collective responsibility and a desire to protect vulnerable groups, rather than personal incentives. Together, these insights underscore the value of aligning new technologies with community expectations, ensuring respectful engagement, and reinforcing shared responsibility in dengue prevention efforts."

48. Comments: In general, the article's significant findings support the importance of aligning technological innovation with local social dynamics. However, there are parts, as stated in the report, that need further depth of analysis and clarification for logical consistency.
Response: We sincerely appreciate the reviewer's insightful comments. In response, we have carefully revised the manuscript to enhance clarity and logical consistency. Specifically, we have addressed the 47 detailed comments noted above, incorporated one additional table to strengthen the presentation of findings, and expanded explanations within the Methods and Results sections. These revisions provide greater analytical depth and ensure that the narrative is coherent and logically aligned throughout the manuscript.
