# Supplementary material for: Community participation and technological innovation: Baseline qualitative insights to inform a five-year cohort on drone-based dengue surveillance in Malaysia
Source: PLoS Negl Trop Dis. 2026 Mar 12;20(3):e0013979. doi: 10.1371/journal.pntd.0013979 (PMC12998943; doi:10.1371/journal.pntd.0013979)
Supplement: Attachment — Submitted filename: PLOS NTD Review - Copy.docx [file pntd.0013979.s001.docx]

**PLOS NTD**

The manuscript “Community Participation and Technological Innovation: A Prospective Cohort Study on Drone-Based Dengue Surveillance” applies a qualitative approach to explore householder and stakeholder perceptions of drone-based surveillance in Selangor. Authors conducted semi-structured interviews with 480 households to elucidate common themes on the use of these drones in residential areas.

The manuscript is well written and the outcomes of the interviews are clear and findings important for improving the future efficiency of container surveillance in areas where dengue is endemic. The authors emphasise that drone-based surveillance is defined by local social and cultural context which is an important finding for these types of potentially invasive technologies. While I can find no major issues with the manuscript, I would like the authors to reconsider some of their language to ensure the reader is not confused about the use of drones for container surveillance.

While there are no lines that I can directly reference in the manuscript to edit, I will use general terms for where I think improvements can be made.

**Use of Terminology**

Throughout the manuscript the authors continually refer to the use of drones for dengue control or dengue surveillance. It is even in the title of the manuscript. The drones being described in this manuscript and references provided are not being applied in dengue control, they are being employed in container surveillance for mosquito vectors of dengue (sometimes referred to as mosquito breeding site monitoring in the manuscript). It is important that this difference is made clear throughout the majority of the manuscript and removed from the title. The only way drones can be talked about in the context of dengue, is to improve outcomes of dengue management or dengue control (ie. improved efficiency of surveillance improves management outcomes).

Use of the term drone interventions and drones as mosquito control tools implies that the drones are acting to manage dengue, when in the case described in the manuscript or in the references they are being used for passive monitoring. Again, please consider the use of a passive term such as surveillance.

**Introduction**

It would benefit the manuscript for readers to know more about the program under which the drones were being used for surveillance in Selangor. What are the objectives of the larger study? To improve the efficiency of mosquito control? Is container surveillance being validated like was done in the studies referenced?

**Findings**

Please provide more context for the readers on what communities you are referring to. Malaysian communities? Findings will be relevant to the culture in which they are being studied, so this needs to be emphasised throughout, especially in the discussion. It is also a limitation to findings which needs to be discussed in the limitations section.

Page 10: please explain in further detail how drones can be deployed with the specific objective of “identifying new clusters based on reported dengue cases” or otherwise remove.

Page 10: You mention drone use “framed around rapid detection and targeted response”. The majority of what has been discussed to this point has been around surveillance. In this context, what do you mean by rapid detection and response?

**Discussion**

Second paragraph needs the font corrected and integrated into one of the other paragraphs before or after this one sentence.

**Limitations**

The use of the wording “potential breeding sites” is critical in this manuscript, as drones are not as useful as active monitoring when samples are taken from containers for identification. Potential mosquito breeding sites still need to be validated by field staff on the ground. Detection of potential breeding sites will be more efficient in dengue endemic areas to direct mosquito control but not for those outside endemic regions that do not utilise insecticide regular spraying programs.

Please include the limitation that findings may only be applicable to the cultural setting they were observed in.

**Conclusion**

Please include Malaysia as the country in which the study was undertaken, after the use of Selangor.

**Specific Issues**

Figure 2: fonts are too small to read.

First use of *Aedes aegypti* in full then use *Ae. aegypti* both with italics.
